# VEGF-D Dependent IFNγ Production by Natural Killer Cells in Lymphangioleiomyomatosis

**DOI:** 10.33696/immunology.7.242

**Published:** 2025

**Authors:** Andrew R. Osterburg, Morgan A Terry, Danielle S. Stiene, Racheal Foot, Rebecca L Nelson, Michael T. Borchers

**Affiliations:** 1 University of Cincinnati College of Medicine, Division of Pulmonary, Critical Care and Sleep Medicine, Department of Internal Medicine, PO Box 45267-0564, Cincinnati, OH 45267; 2 Oregon Health & Science University, Portland, Multnomah County, Oregon, United States

**Keywords:** Natural Killer cells, Phosphorylation, VEGFR3, Lymphangioleiomyomatosis, Cytokines, IFNγ, pSTAT4, pERK1/2

## Abstract

Lymphangioleiomyomatosis (LAM) is a rare cystic neoplasm that almost exclusively affects women. LAM arises from inherited or spontaneous mutations in *TSC1* or *TSC2* genes, which result in constitutive activation of mTOR and downstream signaling. These mutations arise in a uterine population of cells that migrate to the lungs where they establish nodules that progress to cysts and progressive lung function decline. Clinically, an elevated level (>800 pg/ml) of vascular endothelial growth factor -D (VEGF-D) is a definitive diagnostic marker for LAM. Recent work by our group and others highlights the role of immune dysfunction in the establishment and progression of LAM. A subset of natural killer (NK) cells in LAM express the VEGF-D receptors, VEGFR3 and NRP2. These distinct NK cells are responsive to VEGF-D treatment and LAM NK cells have altered NKG2D and CD160 expression, as well as changes in phosphorylation of STAT4(Y693) and ERK1/2 (Y202/S204). Phosphorylation of these proteins increases after VEGF-D treatment at short time points (0-30 min) and longer time points (up to 6 hours). In addition, the signaling kinetics are altered compared to cytokine stimulation. Increased IFNγ production was shown by ELISA and flow cytometry after VEGF-D, and other cytokines. Baseline IFNγ expression is higher in LAM NK cells, and LAM NK cells elaborate IFNγ when stimulated with VEGF-D. Additionally, these NK cells have increased responsiveness to IL12/33 and decreased responsiveness to IL12/IL27 co-stimulation. These data demonstrate that NK cells from LAM patients are functionally altered and these changes in baseline function may contribute to disease progression and pathology.

## Introduction

Lymphangioleiomyomatosis (LAM) is a rare cystic neoplasm that almost exclusively affects women [1,2]. Inherited or spontaneous mutations in the *Tuberous Sclerosis Complex 1 or 2 (TSC1/2)* genes give rise to inactivating mutations in these proteins. TSC1/2 represses mammalian target of rapamycin (mTOR), and mutations in these genes result in constitutive activation of mTOR. This dysregulation promotes cell proliferation, enhances survival, and alters cellular metabolism in mutant cells. In LAM, the affected cells have been identified as smooth muscle-like cells. These transformed cells establish themselves in the lungs and other tissues. In the pulmonary environment, these cells proliferate extensively forming nodules and drive progressive cystic destruction of the lung parenchyma by unknown mechanisms. Clinicians primarily treat LAM with mTOR inhibitors such as sirolimus (rapamycin) or everolimus. Notably, this pharmacologic intervention reduces or halts disease progression in most cases. However, because sirolimus is cytostatic, cessation of therapy results in the resumption of disease progression.

Multiple studies have established that LAM cells originate from an extrapulmonary tissue source. *TSC1/2* mutant smooth muscle-like cells metastasize to the lung. For patients with end stage disease, lung transplantation is the only remaining option. However, cases of LAM recurrence in transplanted lungs provide strong evidence for an extrapulmonary source for LAM cells [3,4]. Data further suggests that LAM cells originate from uterine or neural crest-derived tissues [5]. Recent single-cell RNA sequencing (scRNA-seq) studies strongly support this model and demonstrate that LAM cells isolated from the lung have transcriptional profiles strikingly similar to uterine smooth muscle cells [6]. Furthermore, recent work in our laboratory supports these findings by demonstrating the presence of a LAM-like cell population in a mouse model of LAM [7]. In this mouse model of LAM, we observed that depletion of natural killer (NK) cells, a cytotoxic lymphocyte, resulted in increased lung nodules. Moreover, these nodules occurred at earlier timepoints compared to control LAM mice without the NK cell depletion. Previously, we reported a significant decrease in both the percentage and surface expression of NKG2D on immature (CD56^++^/CD16^−^) and mature (CD56^+^/CD16^+^) NK cells in LAM patients. Consistent with a role in LAM pathogenesis, we also observed reduced expression of the NK cell activating receptor NKG2D (CD314) on peripheral NK cells in LAM patients. Furthermore, a subset of LAM patients exhibited elevated levels of the soluble NKG2D ligands ULBP2 and ULBP3. The presence of these soluble ligands may suppress NK cell cytotoxicity, thereby enhancing disease progression [8]. In agreement with our findings, Liu *et al*. demonstrated a correlation between higher serum IL18 levels and better lung function in LAM patients [9]. IL18 is a well-established NK cell activator and likely contributes to this protective effect. Together, these findings underscore the underappreciated role of NK cells in LAM pathogenesis.

Clinically, a key diagnostic indicator of LAM is a serum vascular endothelial growth factor D (VEGF-D) level greater than 800 pg/mL [10,11]. Elevated VEGF-D in LAM may influence the function of cells expressing receptors for this growth factor. VEGF-D signals through three receptors: VEGFR3 (FLT4), VEGFR2, and neuropilin-2 (NRP2), which play central roles in lymphangiogenesis and lymphatic maintenance [12-14]. Although the literature on VEGFR3/FLT4 in NK cells is limited, VEGFR3^+^ NK cells have been described in acute myeloid leukemia [15]. In AML, FLT4^+^ NK cells exhibit impaired cytotoxicity and fail to eliminate leukemic cells effectively. Treatment with a peptide derived from FLT4’s intracellular domain restored NK cell cytotoxicity, increased T and NK cell numbers, and IFNγ production [16]. In macrophages, FLT4 signaling recruited AMP-activated protein kinase (AMPK) and induced phosphorylation cascades [17]. These findings suggest that FLT4 expression has functional consequences in several disease contexts and immune populations. Based on these data, we investigated whether NK cells from LAM patients express FLT4 and if this expression influences NK cell function.

## Materials & Methods

### Human samples

LAM patients and healthy female volunteers donated fresh whole blood at the University of Cincinnati Pulmonary LAM Clinic after obtaining informed consent (IRB 2013-8157).

### Cell lines

NK-92 MI (CRL-2408) cells were obtained from ATCC, and cultured in α-MEM supplemented with horse serum (12.5%), FBS (12.5%), folic acid (0.02 mM), myo-inositol (0.2 mM), BME (0.1mM), Pen/Strep (1%), L-glutamine (2 mM), NEAA (0.2 nM) at 37C with 5% CO_2_. NK-92 MI VEGFR3 cells were maintained in the same. The generation of the NK-92 MI cell line with VEGFR3/FLT4 stable overexpression was performed by Vector Builder (Chicago) as a service. Cells were transfected with lentivirus pPB[Exp]-Puro-EF1A expression vector and co-transfected with hFLT4[NM_182925.5]:IRES:EGFP.

### PBMC isolation

Peripheral blood mononuclear cells (PBMC) were isolated immediately after blood withdrawal by density gradient centrifugation. Whole blood was mixed 1:1 with 1x HBSS and then placed in Leukosep tubes (Greiner One) prefilled with Lymphoprep (Stemcell Technologies). Samples were then centrifuged for 25 minutes at 1,200 x g with no brake. The PBMC layer was removed and washed twice in Ca^2+^/Mg^2+^ free 1x PBS, followed by RBC lysis with ammonium chloride based 1x lysis buffer (Biolegend). Cells were then washed 2x in cold RPMI, counted and resuspended in cryopreservation media (final 40% FBS, 10% DMSO) and placed in a Mr. Frosty at −80C. Cells were transferred to liquid nitrogen vapor phase within a few days of freezing. When cells were needed the cryopreserved sample was quick thawed at 37C and the sample washed twice in 10ml of RPMI (400 x g, 10 min). Cells were then counted and resuspended in RPMI1640, 10% FBS at an appropriate concentration for downstream experiments.

### Flow cytometry staining

Samples were analyzed on an Attune (Invitrogen) 2-laser flow cytometer equipped with 488 nm and 635 nm lasers and 4 blue and 2 red fluorescence detectors. Cells were gated first on FSC-A versus SSC-A and then FSC-A versus FSC-H to exclude doublets. Viable cells were also selected after staining with a fixable live/dead reactive ester. Some Samples were stained and analyzed on a 5-laser Aurora Cytometer (Cytek). Phospho staining employed the following antibodies. pSTAT4 Y693 (PE: clone 38), anti-pSTAT5 (Y694) (PE: clone 47), anti-pERK1/2 (pT202/pY204) (APC: clone 20A) were purchased from BD Biosciences. Anti-human CD3 (PerCP-eFluor710: UCTH1), CD56 (KIRAVIA Blue: 5.1H11), CD16 (PE-Cy7:clone 3G8), FLT4 (PE: clone 9D9F9) and Zombie Fixable NIR Viability dye were obtained from (Biolegend). NRP2 (Alexa 647: Clone 257103) was purchased from RnD systems. For each sample, at least 10,000 viable cells of interest were collected. Data was analyzed with Kaluza (v2.3). Annexin V-APC and propidium iodide were obtained from BD biosciences. Cells were treated as described with various concentrations of VEGF-D overnight before staining for annexin V/PI. After treatments cells were washed twice in cold PBS (400 x g, 10 min). Subsequently, cells were resuspended in 1x binding buffer and AnnexinV and PI added. PI was used at 1 μg/ml to test for membrane permeability. Cells were analyzed immediately by flow cytometry.

Intracellular staining (ICS) was performed on treated cells following experimental conditions. Immediately after treatment, cells were fixed in a final concentration of 2% paraformaldehyde (Electron Microscopy Services) for 25 minutes at room temperature. Cells were washed once with flow buffer (FB), and then 2 ml of ice-cold methanol was added while vortexing to the residual FB in the tube for a final concentration of 90% MeOH. Cells were then stored at −80C until staining. For staining, cells were rehydrated by washing twice with FB (400 x g, 10min). After the second wash, cells were resuspended in ~100 μl of FB and blocked with nonspecific mouse γ-globulins (Sigma-Aldrich) for 20 minutes at room temp. Subsequently, appropriate phospho-specific antibodies were added for 30 minutes in the dark at room temperature. Cells were washed twice with FB and analyzed by flow cytometry. Compensation controls were prepared with Ultracomp beads (ThermoFisher) as needed. Surface staining was performed on isolated cells after experimental treatments. Cells were washed once with cold 1x PBS and resuspended in 100 μl of the same. Zombie NIR was added, and cells were incubated on ice for 20 minutes in the dark. Samples were then washed twice in cold FB. Afterwards samples were blocked with TrueStain Fcx (Biolegend) for 20 minutes on ice and then appropriate antibodies added for 30 minutes, followed by two washes FB. Cells were fixed with 1% PFA and analyzed within 24-hours.

Samples were run and analyzed on the Aurora and were stained as per Whyte *et al*. [18]. Briefly, cells were treated for experimental conditions as needed. Live cells were then labelled with NIR fixable live/dead (Invitrogen) reactive ester, on ice, and in the dark. After two washes with cold FB, cells were then fixed with 0.2% paraformaldehyde for 25 minutes at room temperature in the dark. Cells were then washed twice with FB, resuspended in ~60-80 ul of FB and blocked with 5 ul each of Human TruStain FcX and True Stain Monocyte Blocker (Biolegend) for 25 min at room temperature in the dark. Antibodies were added to appropriate samples and incubated overnight at room temperature in the dark. Cells were washed, filtered through a 40 um mesh tube top filter, and analyzed on a 5-laser Aurora (Cytek). All antibodies used were titrated for optimal staining concentrations. Additionally, staining cocktail included 1x Brilliant Violet Stain buffer. FMO’s were employed to set positive and negative gates. Unmixing controls were made with Spectracomp or SpectracompXT beads and stained under the identical conditions to the samples. Data was unmixed in Spectroflow software (v3.3).

### Specific lysis

Specific lysis was assessed by incubating NK cells with Jurkat cells (E6-1, ATCC) as target cells. Briefly, PBMCs were rapidly thawed, washed in complete RPMI and resuspended in a cell culture plate with 40U/ml IL2 (Peprotech). After 24 hours, CD56^+^ cells were negatively selected using magnetic beads (Stemcell Technologies). NK cells were counted and placed in with an appropriate quantity of target cells. Effector and target cells were incubated in round bottom 96-well plates for 4 hours, followed by washing and staining with Annexin V/PI. NK cells and Jurkat target cells were separated due to distinct FSC-A and SSC-A profiles of the cell types. Spontaneous cell death was determined by incubating target cells alone, while maximum lysis was induced by treating targets with 10% EtOH for 10 minutes. Following incubation, cells were immediately analyzed by flow cytometry. Percent specific lysis was calculated using the formula: ((% PI^+^ experimental - % PI^+^ spontaneous)/(% PI^+^ maximum - % PI^+^ spontaneous)) × 100.

### ELISA

ELISA kits for IFNγ, sVEFGR3/sFLT4 were obtained from Bio-Techne. ELISAs were performed according to manufacturer’s protocols. Serum was diluted 1:4 or 1:8, depending on the available sample volume.

### VEGFR3

To validate the specificity of the mouse anti-human VEGFR3-APC antibody we performed blocking experiments using a 10-fold excess of unconjugated anti-VEGFR3 antibody prior to APC-conjugated antibody. Significant staining was observed with 5 or 2.5 µL of antibody, and preincubation with a 10× excess of the unconjugated antibody reduced staining to levels comparable to the no-antibody control (Supplemental Figure 1A).

### Statistics

Statistical significance taken at p<0.05, Where appropriate corrections were made to correct for multiple tests. Student’s t-tests were performed with Prism Software (Siemens).

## Results

### Expression of VEGFR3/FLT4 and soluble VEGFR3 from lymphangioleiomyomatosis patient NK cells

**Figure 1.**
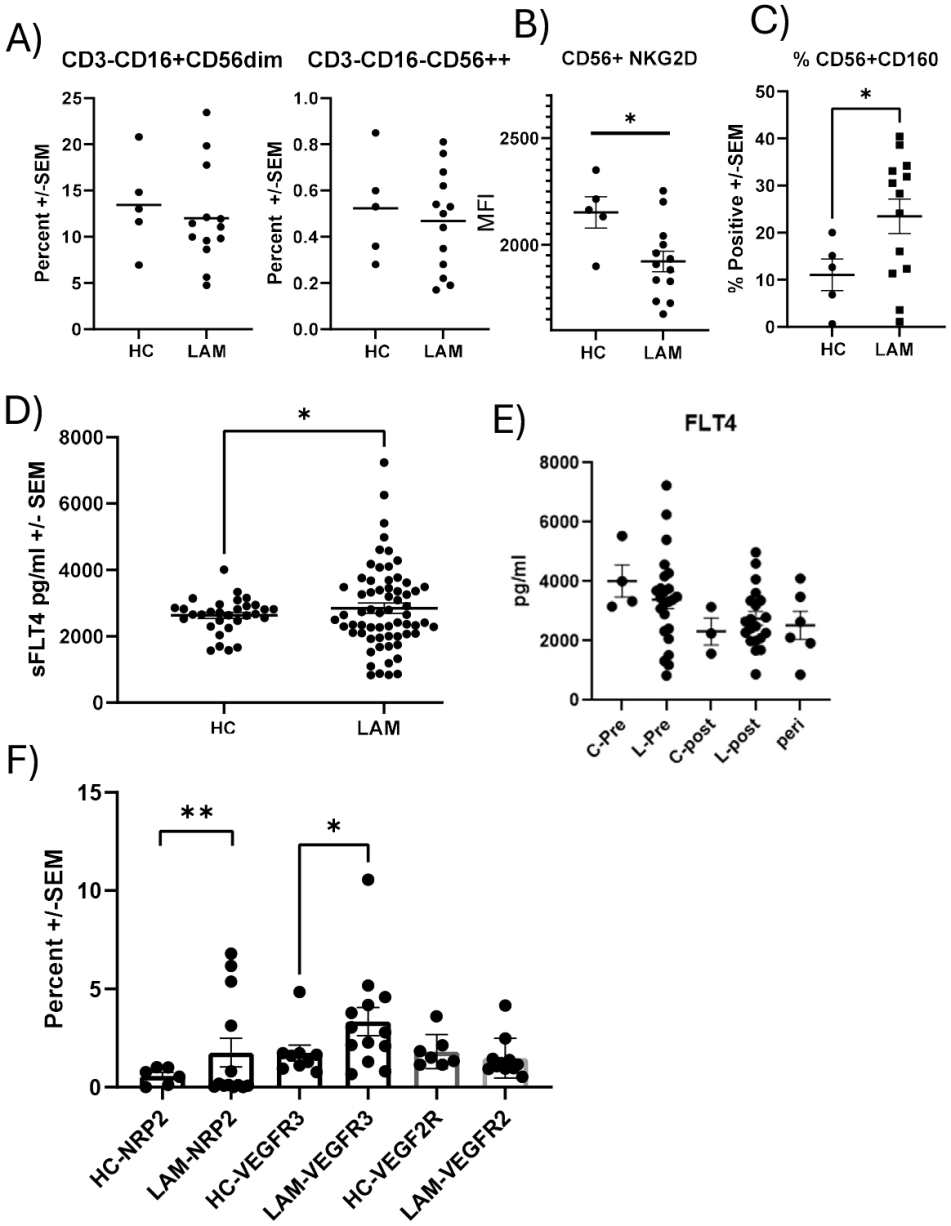
Soluble VEGFR3(FLT4) and Surface Expression of VEGFR3 on NK cells. **A)** Phenotyping the abundance of CD3^−^CD16^+^CD56^dim^ and CD3^−^CD16^−^CD56^++^ NK cells in the peripheral blood of healthy controls and LAM patients, n=5–13. **B)** The Mean fluorescent intensity (MFI) of NKG2D on control and LAM CD56+ cells from peripheral blood, n=5–13. **C)** Increased CD160 surface expression on CD56+ LAM cells from peripheral blood, n=5–13. **D)** Expression of sVEGFR3 is increased in sera of LAM patients vs. controls by ELISA, n=31–63 *P<0.05. **E)** sVEGFR3 plotted for healthy controls (HC) and LAM patients based on menopausal status, n=2–20. **F)** Surface expression of NRP2, VEGFR2, and VEGFR3 was measured by flow cytometry on NK from healthy controls and LAM patients, n=6–20. Error bars represent mean ± SEM. *p<0.05, **P<0.01.

Our initial investigation on the role of NK cells in LAM focused on soluble ligands of NKG2D receptors in serum, which correlated with lung function decline [8]. Consistent with these observations we did not observe differences in the frequency of CD3^−^CD16^+^CD56^dim^ or CD3^−^CD16^−^CD56^++^ NK cells between healthy controls and LAM patients (Figure 1A). However, in line with our earlier data, we did observe significantly reduced NKG2D surface expression on CD56^+^ cells (Figure 1B). Notably, LAM patients expressed significantly more Natural Killer Cell Receptor BY55/CD160 compared to controls on CD56^+^ cells (Figure 1C). Due to the presence of soluble ligands for NKG2D we measured the presence of soluble VEGFR3 (sVEGFR3) in sera from healthy controls and LAM patients. We found significantly elevated quantities of sVEGFR3 in LAM patients compared to healthy controls (Figure 1D). Stratification of data by menopausal status revealed that the quantity of sVEFGR3 was higher in premenopausal women (Figure 1E). Because of differences in soluble VEGFR3 we then measured surface expression of VEGF-D receptors (VEGFR3, NRP2, VEGFR2) on NK cells from healthy controls and LAM patients. We determined, by flow cytometry, there was significantly more NRP2 (p<0.01) and VEGFR3 (p<0.05) on LAM patient NK cells compared to healthy controls (Figure 1F). VEFGR2 is another receptor for VEGF-D, but we did not observe differences in surface expression. In representative high dimensional flow cytometry staining of LAM cells, we saw expression of VEFGR3 in NK cells (Supplemental Figure 1). CD3^−^CD14^−^ CD16^+^CD56^−^ NK cells had the largest proportion of VEGFR3, with CD16^+^CD56^+^CD57^+^ NK displaying a modest expression of the receptor. Other subpopulations of NK cells displayed little VEGFR3. VEGFR3 expression is not commonly reported in the literature, therefore we used excess unlabeled isoclonic antibody (Supplemental Figures 2A-2E) to determine if the staining was specific. We only see significant staining without unlabeled VEGFR3 antibody. The percentage of positive and median fluorescent intensities were similar to healthy controls. We examined if there were any effects of rapamycin or rapalog treatment on the expression of VEGFR3 on NK cells and found no significant differences. Additionally, we tested if the soluble VEGFR3 was affected by mTOR inhibition. We found no significant changes between patients not receiving or on rapamycin (Supplemental Figures 2D-2E).

### LAM NK and NK92-MI cells apoptosis and specific lysis were similar to control NK cells

**Figure 2.**
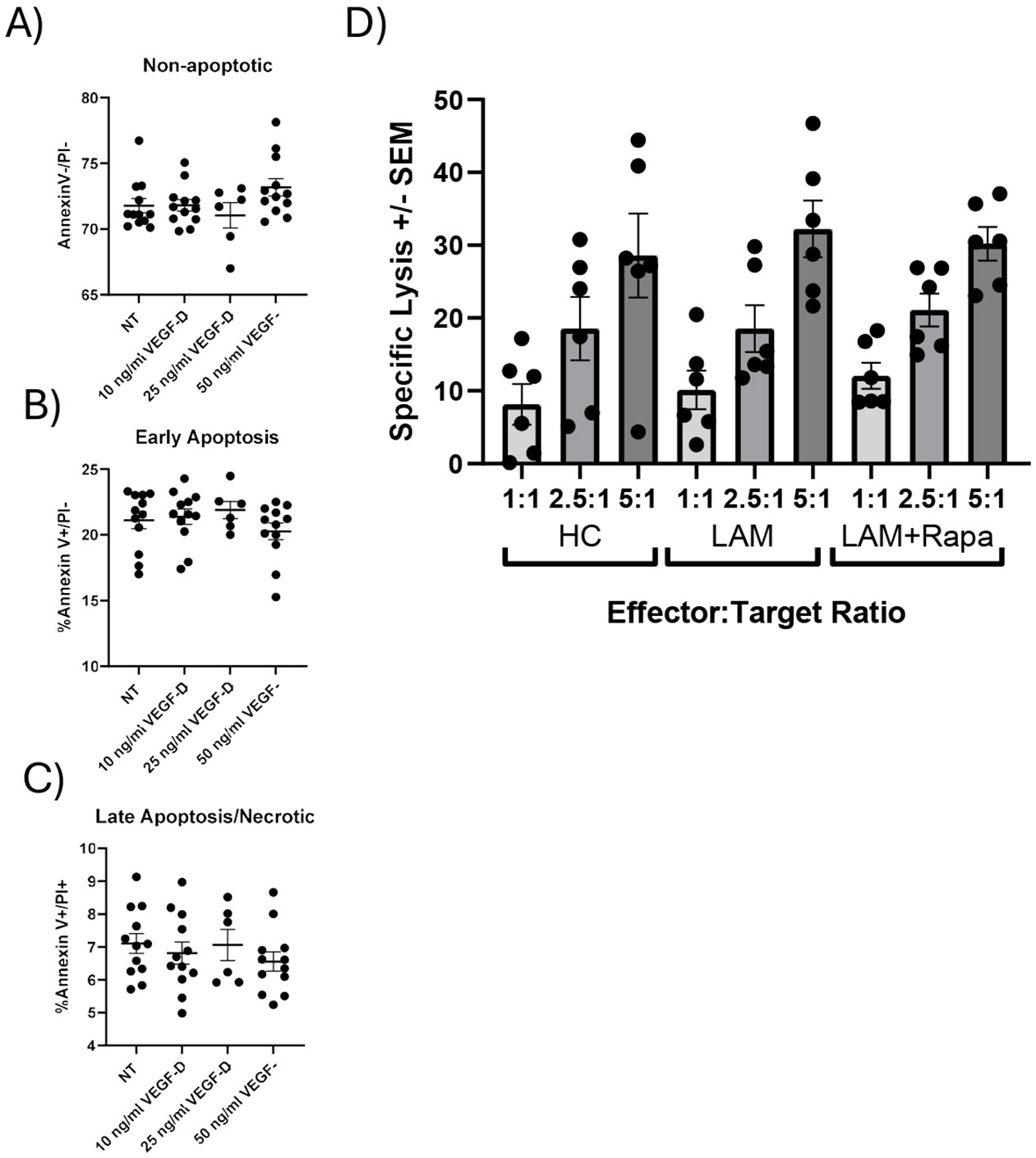
Functional characterization of NK cells from healthy controls and LAM patients. The effects of VEGF-D treatment (10-50ng/ml) for 24 hours prior to staining with annexin V (AV) and Propidium Iodide (PI) was used to detect changes in early and late apoptosis/necrosis. **A)** shows non apoptotic cells as defined by AV- and PI- staining. **B)** Early apoptosis (AV+/PI−) shown for cells treated with VEGF-D. **C)** Late apoptosis/early necrosis (AV+PI+ or AV−PI+). n=12 per treatment. **D)** Specific lysis of NK cells from healthy control and LAM patients was performed at various effectors to target ratios. No significant differences between HC and LAM NK cells were observed, n=6 per group. Error bars represent mean ± SEM.

We next assessed if increased VEGFR3 expression on NK cells had functional consequences. We first examined if VEGF-D treatment (NT, 10, 50 ng/ml of VEGF-D) on cultured NK92-MI cells, an IL2 independent immortalized NK cell line, to determine if VEGF-D altered apoptosis. We stained cells for annexin V and propidium iodide (Figure 2). We found no significant differences in the proportion of viable (annexin V^−^/PI^−^) (Figure 2A), early (annexin V^+^/PI^−^) (Figure 2B), or late apoptotic/necrotic cells (annexin V^+^/PI^+^) (Figure 2C). Additionally, we examined specific lysis of NK cells from healthy controls and LAM patients. As shown in Figure 2D, no significant differences across various effector-to-target ratios between groups were noted. Furthermore, NK cells from patients currently under rapamycin/sirolimus did not show altered cytolytic activity (Figure 2D).

### LAM NK cells have distinct differences in phosphorylation compared to controls

**Figure 3.**
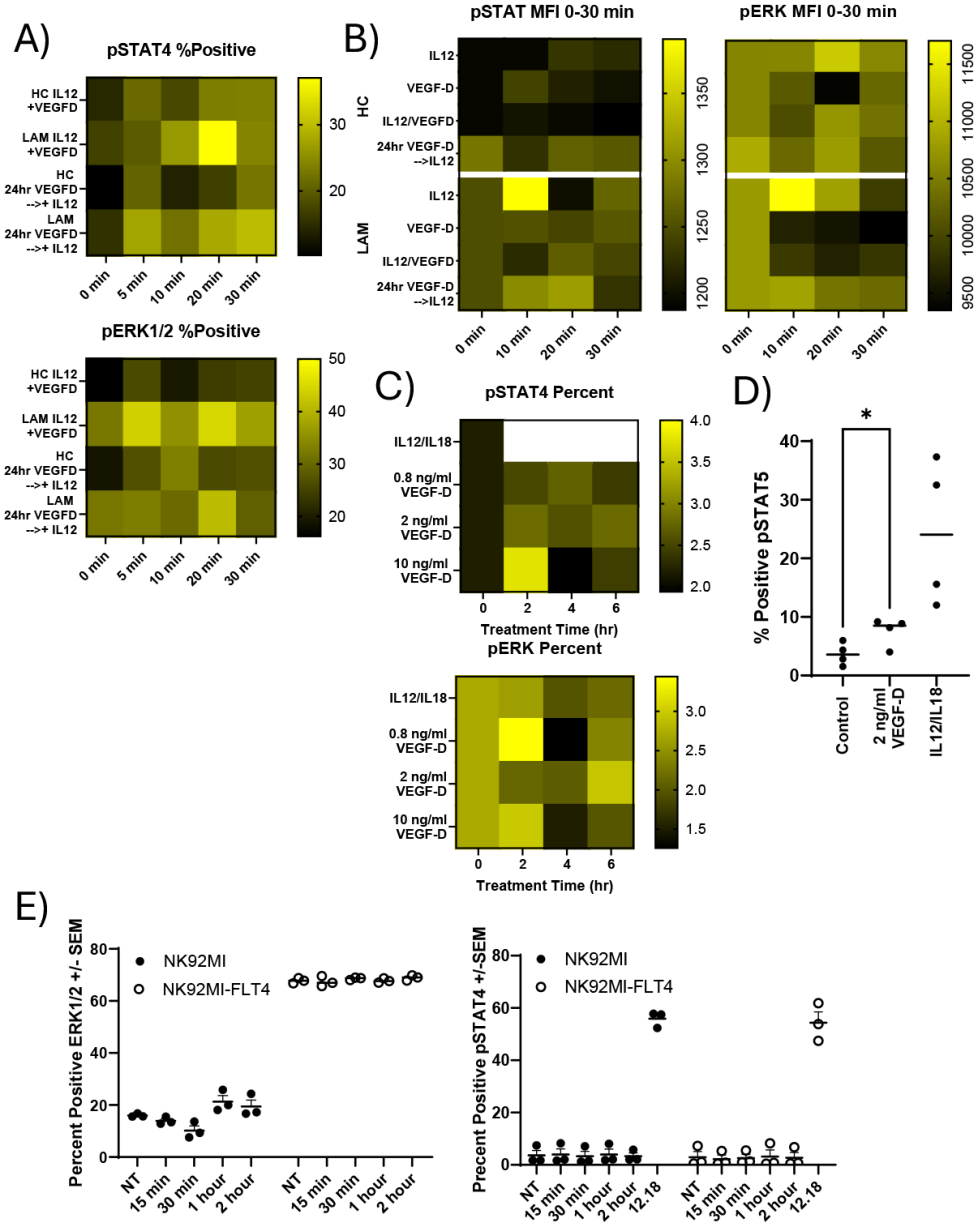
Phosphorylation changes in NK cells from HC and LAM patients. **A)** NK cells were isolated from peripheral blood of LAM patients and healthy controls by positive magnetic bead selection. Cells were cultured overnight in SCGM-complete, 240U of IL2, 10% AB Serum, and treated with 100ng/ml of VEGF-D, IL12 and IL18 was completed as a time course ranging 0–30 minutes. Cells were processed for intracellular staining for phosphorylation. Cells were analyzed by flow cytometry to determine percent positive for pSTAT4 (Y693)-APC and pERK1/2(T202/Y204)-PE. Graphs are representative of phosphorylation changes in NK cells. **B)** Heatmaps of the MFI of NK cells from Healthy controls and LAM patients. **C)** NK cells were treated as above for up to 6 hours. After the treatment period, cells were processed for intracellular staining as described. All heatmaps are representative. **D)** NK cells were stained for pSTAT5 after treatment with VEGF-D or IL12/18 for 30 minutes, n= 4/group. **E)** percent positive for pSTAT4 and pERK1/2 in NK92MI cells stably transfected with VEGFR3. n= 3/group, P>0.05.

Previous work on LAM NK cells revealed changes in surface expression of NKG2D, among other changes. Therefore, we examined if any IFNγ signaling changes in phosphorylation cascades were present in LAM NK cells. The effects of VEGFR3 signaling on the phosphorylation status of STAT4 (pY693) and ERK1/2 (T202/Y204) in NK cells isolated from LAM patients and healthy controls were examined. NK cells were treated with and without VEGF-D, IL12, IL18 for varying times. Cells there then fixed and stained intracellularly for pSTAT4 and pERK1/2. As shown in Figure 3A, LAM NK cells displayed distinct phosphorylation responses compared to controls, particularly following treatment with VEFG-D and IL12 for up to 30 minutes. In LAM NK cells there was an increase in STAT4 and ERK1/2 percent positive cells. In these shorter time courses, there was also an increased median fluorescent intensity (MFI) of pSTAT4 in LAM patients. Likewise, pERK1/2 in LAM patients NK cells were more responsive to IL12 stimulation than controls (Figure 3B). Exposure to IL12 increased pSTAT4 phosphorylation at 10 minutes which was markedly blunted by 24-hour pretreatment with VEGF-D. Likewise, in healthy patients, VEGF-D modestly altered the MFI of pSTAT4. In terms of responsiveness, IL12 treatment of LAM cells are more responsive to VEGF-D than healthy controls. These trends of higher phosphorylation also appear consistent with pERK1/2 in LAM samples compared to healthy controls. In extended VEGF-D treatments (2-6 hours) we observed significantly altered phosphorylation kinetics of both pSTAT4 and pERK1/2 in a VEGF-D dose-dependent manner (Figure 3C). VEGF-D treatment in LAM NK cells also resulted in increased STAT5 phosphorylation (Figure 3D). There was approximately a 2-fold increase in positive pSTAT5 cells after VEGF-D exposure compared to controls. To further examine these mechanisms, we transfected NK92MI cells with a stable expression vector expressing VEGFR3. As shown in Figure 3E there was a dramatic basal increase in pERK1/2 phosphorylation in the transfected cells. In contrast, changes in pERK1/2 were modest in the control cells. In these transfected cells, phosphorylation of STAT4 appeared to be unaffected.

### VEGFD affects signaling phosphorylation and increases cytokine production

**Figure 4.**
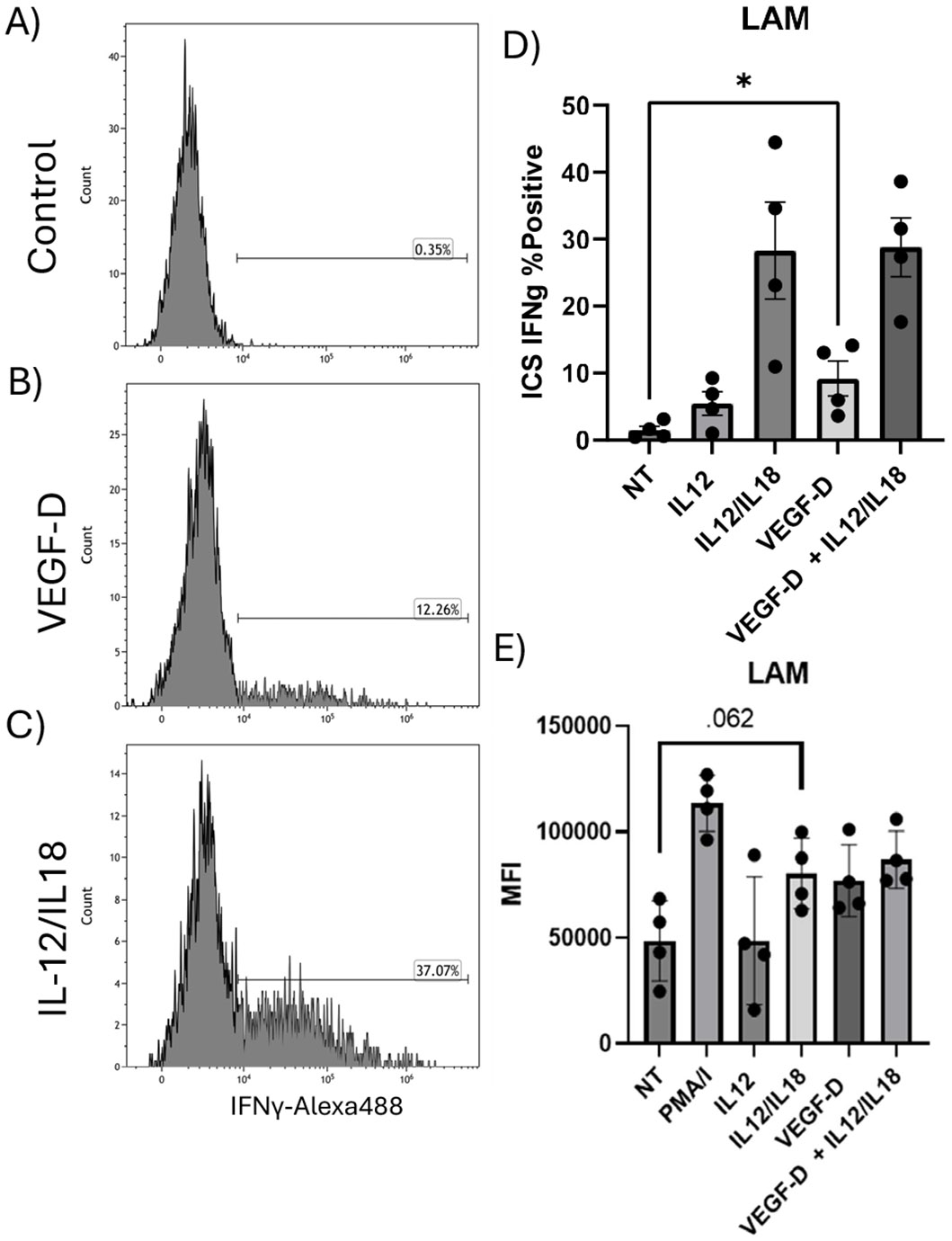
Intracellular staining for IFNγ of NK cells from HC and LAM patients. **A-C)** Flow cytometry histograms of IFNγ-Alexa488. Cells were control **(A)**, treated with VEGF-D **(B)**, or IL12/IL18 **(C)**. During exposure cells were exposed to BrefeldinA and after treatment cells were fixed and processed for intracellular detection of IFNγ with staining for phenotyping markers as well as VEGF-D receptors. Quadrant gates were set with appropriate FMO controls. Histograms are representative. **D)** Stimulation of IFNγ expression in NK cells from LAM patients after various treatments. Plot shows the percent positive of IFNγ staining by ICS. NT vs. VEGF-D, *p<0.0. **E)** Median fluorescent intensity (MFI) of the ICS for IFNγ. Data shows mean ± SEM. n= 4/group.

Due to VEGF-D dependent phosphorylation in STAT4 and ERK1/2, we next examined if there were changes in IFNγ production in NK cells treated with VEGF-D and other pro-inflammatory cytokines. Therefore, we performed intracellular cytokine staining (ICS) of NK cells for IFNγ cellular content by flow cytometry. Figures 4A-4C shows representative flow cytometry histograms for these treatments. At baseline, NK cells elaborate little IFNγ (~0.35%) (Figure 4a). After addition of VEGF-D there is increased production of IFNγ (~12.26%) (Figure 4b). NK cells treated with IL12/IL18 induced a substantial intracellular IFNγ response (~37.07%) (Figure 4c). VEGF-D treatment also increased the frequency of IFNγ positive cells (p<0.0273) (Figure 4D). Additionally, we noted that there was a corresponding increase in the MFI of IFNγ of VEGF-D treated samples. While not statistically significant (P<0.062) the MFI increase is in line with the other stimulation conditions (Figure 4E).

### Consequences of altered intracellular phosphorylation on IFNγ in cultured NK cells

**Figure 5.**
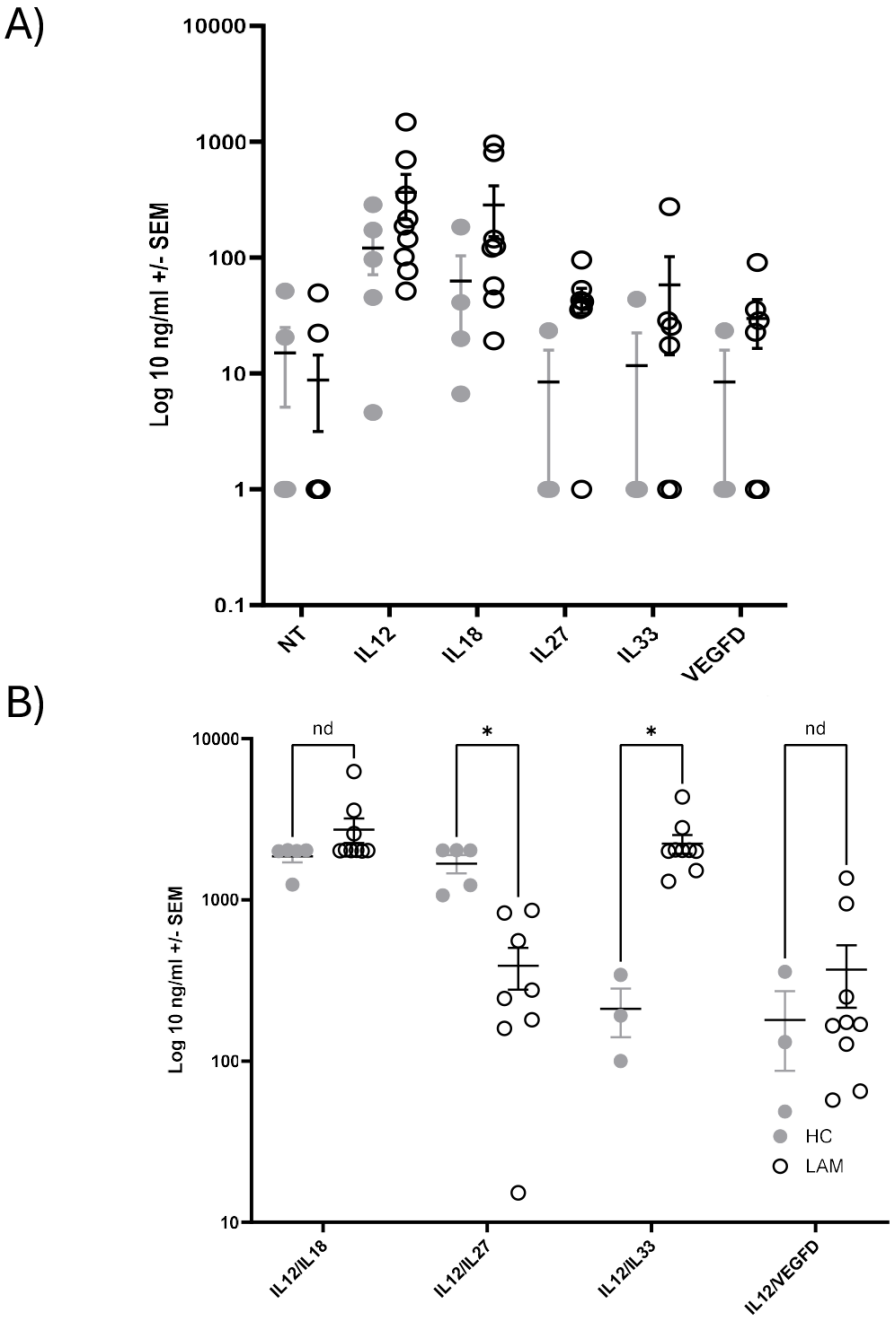
Natural Killer cell production of IFNg from LAM patients after stimulation. NK cells were isolated from peripheral blood of healthy controls and LAM patients. 10,000 cells/well were plated with in SCGM-complete media (240U IL2, 10% AB serum) with and without 20 ng/ml VEGF-D and or IL12 (20 ng/ml)/IL18 (20 ng/ml) for 24 hours. Supernatants were collected and assayed for IFNγ by ELISA. Mean ± SEM. **A)** Plot of single cytokine stimulations. **B)** plot of stimulation with two cytokines. HC (grey circle), LAM (empty circle). n= 4–9/group.

Finally, we measured IFNγ synthesis from cultured NK cells, treated with various cytokines, from LAM patients and healthy controls for 24 hours. IFNγ was assayed from cell culture supernatants by standard ELISA. As shown in Figure 5A, there was increased IFNγ synthesis in LAM patients across all treatments. In particular, VEGF-D increased IFNγ production in LAM NK cells over healthy controls. Consistent with prior studies, there was no synergy between VEGF-D and IL12/IL18. However, stimulation with IL12 and IL33 significantly enhanced IFNγ production in LAM patients (Figure 5B) (p<0.05). In contrast when NK cells were cocultured with IL12 and IL27 there is reduced IFNγ production in LAM patient NK cells (p<0.05).

## Discussion

Our data demonstrate that NK cells from LAM patients are functionally altered by exposure to VEGF-D. Consistent with previous data, we did not observe changes in the abundance of either immature (CD16^−^CD56^+^^+^) or mature (CD16^+^CD56^+^) NK cell subsets in LAM patients compared with healthy controls. In contrast, and in line with our prior findings, we observed alterations in surface receptor expression (NKG2D, CD160) on LAM NK cells that suggest functional differences are present as well. Notably, a small but significant subset of NK cells in LAM patients expressed VEGF-D receptors (NRP2, FLT4). Signaling through these receptors influences downstream phospho-signaling pathways (STAT4, ERK1/2, STAT5), which may contribute to the increased IFNγ synthesis observed in LAM.

VEGFR3 expression on NK cells has not been widely reported and is typically not expressed on CD45^+^ cells. However, in cancer and inflammatory conditions, expression on mononuclear cells has been observed [19,20]. In acute myeloid leukemia (AML), VEGFR3 expression has been described and is correlated with poorer prognosis [15,16]. Increased VEGFR3 expression in AML is linked to NK cell dysfunction. In contrast to our findings, VEGFR3 expression in AML was associated with decreased IFNγ production. In AML, treatment of NK cells with an anti-VEGFR3 peptide restored IFNγ production and improved prognosis. In LAM we see a modest, but significant increase in IFNγ elaboration NK cells. In these studies, IFNγ functions primarily as a marker to assess NK cell activation, representing a well-established indicator for this purpose. Expression of IFNγ in LAM has been shown in a mouse model of LAM [21], and in sera from LAM patients. We show increased CD160 expression in LAM patients. Expression of this receptor suggests activated NK cells and increased IFNγ production. Tu *et al.* demonstrated that CD160 plays a significant role in IFNγ production in NK cells [22]. This is paired with decreased surface expression of NKG2D, suggesting that LAM NK are functionally dysregulated. Decreased NKG2D expression was shown to be predictive of COVID-19 severity. In melanoma patients, decreased NKG2D expression was an indicator of impaired NK cell function and poor prognosis [23].

The consequences of increased soluble VEGFR3 (sVEGFR3) in the context of LAM are unknown. It has been reported that sVEGFR3 sequesters VEGF-C [24], and that the increased presence of sVEGFR3 is associated with malignancy in melanoma [24,25]. In our study, we detected increased expression of VEGF-D receptors on the surface of LAM NK cells. These receptors include VEGFR3, NRP2, and VEFGR2. For VEGFR3 and NRP2 expression, while significant, was restricted to a subset of LAM NK cells. Additionally, we did not detect changes in membrane or soluble VEGFR3 due to rapamycin in these patients. The presence of VEGFR3 on LAM NK cells alters intracellular signaling cascades, as supported by our data from the NK92MI-VEGFR3 cell line. In these cells, VEGFR3 is overexpressed and correlated with dramatically increased ERK1/2 basal phosphorylation. This may reflect alterations of intracellular phospho-signaling scaffolding proteins and/or deregulation of pERK1/2 by sequestration. The mechanisms by which NK cell acquire VEGFR3 expression are unknown. It may result from the unique changes in cell stimulation within the LAM microenvironment. Alternatively, NK might acquire VEGFR3 by trogocytosis [26,27] or via exosomal transfer [28]. Serum levels of VEGF-D (>800pg/ml) is a diagnostic marker of LAM [11,29]. Because LAM NK cells respond to VEGF-D it would seem unlikely that we are detecting sVEGFR3 non-specifically interacting with the NK cells. Moreover, while there was increased sVEGFR3 expression in LAM, there was considerable overlap in the expression levels in healthy controls and LAM. Again, it seems unlikely that the presence of this receptor on LAM NK cells is non-specific. Taken together these data show that phosphorylation networks in LAM NK cells are altered compared to healthy controls.

Functionally, the consequence of these phospho-signaling dysregulations is largely associated with changes in IFNγ production in NK cells. We did not observe changes in apoptosis or in specific lysis of LAM NK cells. Additionally, neither function was altered by rapamycin treatment of LAM patient samples. The impact of increased chronic IFNγ expression in the LAM neoplasm is unknown. It has been demonstrated that low IFNγ expression promotes tumor expansion in several models [30]. Low IFNγ expression is consistent with upregulation of PD-L1. Indeed, PD-L1 has been shown to be upregulated in LAM [31].

We cannot describe the root causative effect of the increased IFNγ that we observe here in LAM and that we observed in a mouse model of LAM [21]. In this murine model of LAM there was significant quantities of IFNγ in the serum of these mice. Notably, the *tsc2* knockout was not present in immune populations, and therefore the cause of the increased IFNγ, still unknown, but is an indirect effect of the *TSC* mutation in other cells. It is unlikely, given the expression levels of VEGFR3, that this receptor is the sole cause of increased IFNγ in these unique NK cells. It is becoming clear that there are notable alterations of immune function in LAM. We and others have shown that changes in the immune system contribute or has been altered by the pathophysiology of LAM. The chronic IFNγ expression is reported to increase resistance of some tumors to NK cell mediated killing [32,33]. We also note that immune alterations are reported in LAM. T-cells in LAM nodules demonstrate evidence of exhaustion with expression of PD-1 on T-cells with PD-L1 expression on macrophages, monocytes, and myeloid derived suppressor cells in the tumor microenvironment of LAM [32]. B7-H3 (CD276) CD4 T-cell suppression is also reported in LAM [34]. Taya *et al*. found that neutrophil elastase expressing myeloid cells were present in the lungs of LAM patients, potentially driving LAM tumor progression [35]. We have shown in the uterus of a mouse model of LAM alterations in the number and phenotype of NK cells and that these cells have increased baseline production of IFNγ. Additionally, we observed a dramatically increased number of macrophages in tissue in the LAM model mice [21]. Taken together, these data show that the immune system is altered in LAM and these changes may promote tumor survival and contribute to disease progression.

IL33 is an alarmin and member of the IL1 cytokine family. Initially described as a Th2 inducer, it also functions as a stress and damage signal. Th2 immune responses can be driven by IL33 activation through its receptor ST2. More recent data indicates that IL33 also stimulates ILC2 cells, as well as CD8+ T cells and NK cells. The receptor for this cytokine is a heterodimer of ST2 and IL1 receptor accessory protein (IL1RAP). Release of IL33 is thought to occur upon damage or necrosis of barrier cells. NK cell responsiveness to IL33 has been reported in both mouse models and humans [36,37]. In CD8 T-cells, IL33 in combination with IL12, stimulate IFNγ expression in these cells [38]. In pulmonary diseases, IL33 and its receptor are significantly upregulated in response to tissue damage in COVID-19 patients [39]. In COPD, in both human disease and mouse models IL33 expression is typically increased in endothelial cells in often associated with poor outcomes [40]. We observed significantly greater IFNγ response in LAM patients compared to healthy.

IL12 and IL27 are part of the IL12 heterodimeric family of cytokines. They are central to IFNγ production in NK cells and T-cells and promote Th1 differentiation. The IL12 family also includes IL23 and IL35. These cytokines signal through heterodimeric receptor complexes through the JAK/STAT partners, and IL12 has been described to involve JAK2/STAT4 while IL27 through JAK1/STAT3. While is it unclear why IL12/IL27 stimulation results in less IFNγ production, it is possible that competition between STAT4 and STAT3 for dimeric partners results in reduced IFNγ production in LAM NK cells. With increased basal IFNγ stimulation in LAM cells, arising from low level chronic IFNγ production, a portion of available intracellular STAT4 is sequestered in the nucleus and may not be available for dimerization and cross-phosphorylation [41].

Synergy between IL12 and IL1 families is thought to be mediated by JAK2/STAT4 interactions. Additionally, inflammatory transcription (NF-κB, AP1) factors are downstream of these STAT and ERK pathways. There is also considerable crosstalk between ERK pathways of IL12 and IL33. Furthermore, Iκbζ is shown to necessary and augment IFNγ expression for both IL12 and IL33. This suggests that there is increased activation and linkage between NFκB due to IL12/33 signaling in LAM NK cells [42,43]. Taken together, LAM NK cells are dysregulated compared to healthy controls.

## Conclusion

The inflammatory milieu of LAM is poorly described in the literature. We have previously shown that soluble NKG2D ligands are increased in the sera of LAM patients and correlate with greater FEV1 loss compared to controls. These data indicate that LAM NK cells are altered in their cytokine responses and that VEGFR3 expression may be associated with these changes in NK cell function. The increased low-level chronic production of IFNγ may impair anti-tumor responses in LAM. These studies demonstrate a unique immune phenotype in LAM that may have downstream effects on the control of disease pathogenesis. Going forward, it will be essential to demonstrate that alterations in IFNγ production and phospho-signaling are associated with increased disease progression. Broadly, we add to the growing body of evidence that functional disruptions of the immune systems in LAM patients may have a larger role in disease progression than previously understood. Additionally, these data of immune dysfunctions in LAM suggest that immunotherapy with therapeutic interventions such as immune checkpoint inhibitors or neutralizing antibodies may be valuable in the treatment of the disease.

## Supplementary Material

JCI-25-242-Supplemental-File
