# Supplementary material for: VEGF-D Dependent IFNγ Production by Natural Killer Cells in Lymphangioleiomyomatosis
Source: J Cell Immunol. Author manuscript; Available in PMC 2026 May 13. (PMC13166106; doi:10.33696/immunology.7.242)
Supplement: JCI-25-242-Supplemental-File [file NIHMS2170945-supplement-JCI-25-242-Supplemental-File.pdf]

Osterburg AR, Terry MA, Stiene DS, Foot R, Nelson RL, Borchers MT. VEGF-D Dependent IFNγ Production by Natural Killer Cells in Lymphangioleiomyomatosis. J Cell Immunol. 2025;7(6):204–215.

Supplementary Figure 1

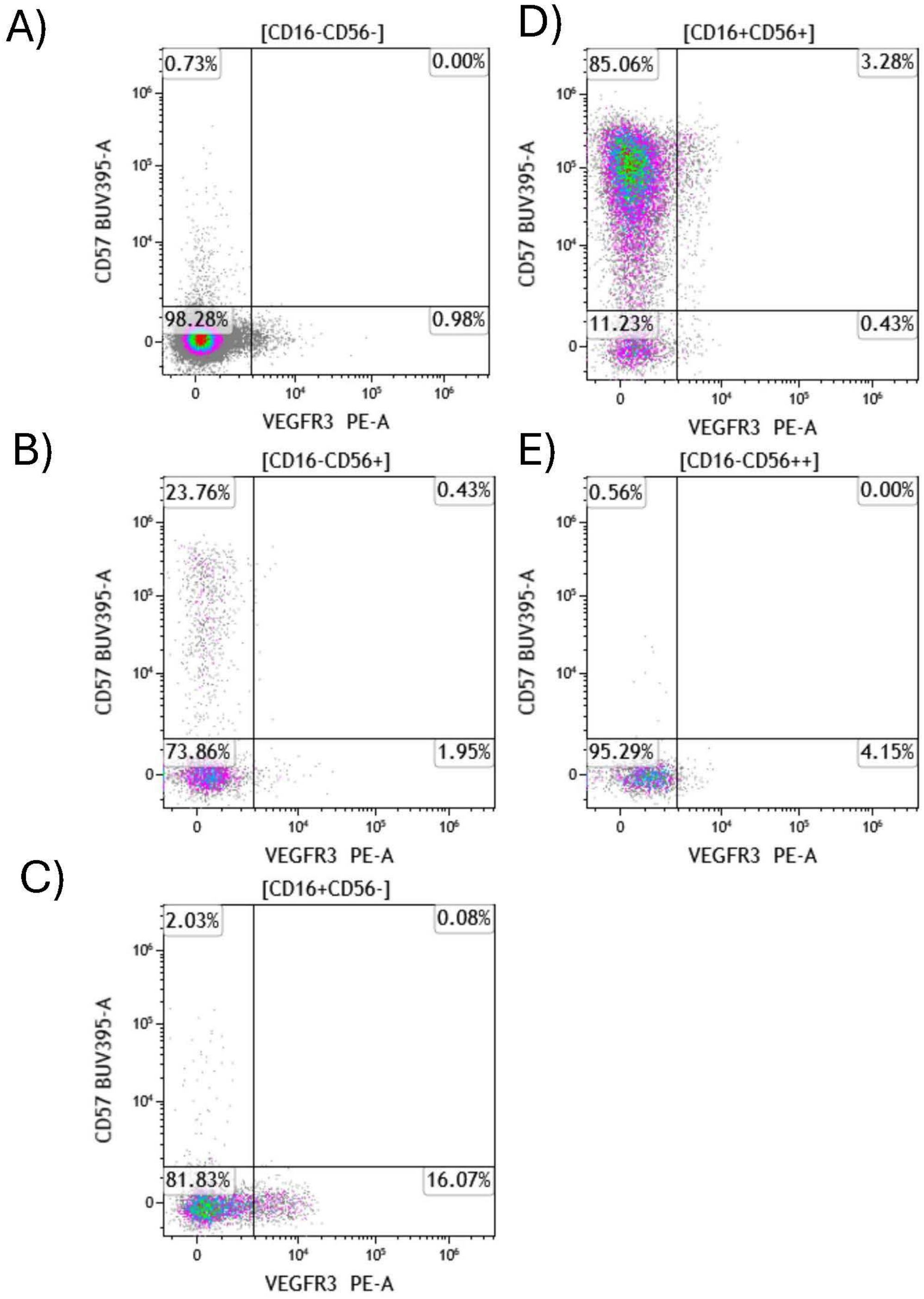

Supplemental Figure 1. VEGFR3 expression on NK cells. Representative flow cytometric scatter plots for detection of VEGF3R-PE and CD57-BUV395 on gated NK cells (Viable, singlets, CD14-, CD3-. The gating of each plot **A)** $CD16^{-}CD56^{-}$, **B)** CD16-$CD56^{+}$, **C)** $CD16^{+}CD56^{-}$, **D)** $CD16^{+}CD56^{+}$, **E)** $CD16^{-}CD56^{++}$.

Osterburg AR, Terry MA, Stiene DS, Foot R, Nelson RL, Borchers MT. VEGF-D Dependent IFNγ Production by Natural Killer Cells in Lymphangioleiomyomatosis. J Cell Immunol. 2025;7(6):204–215.

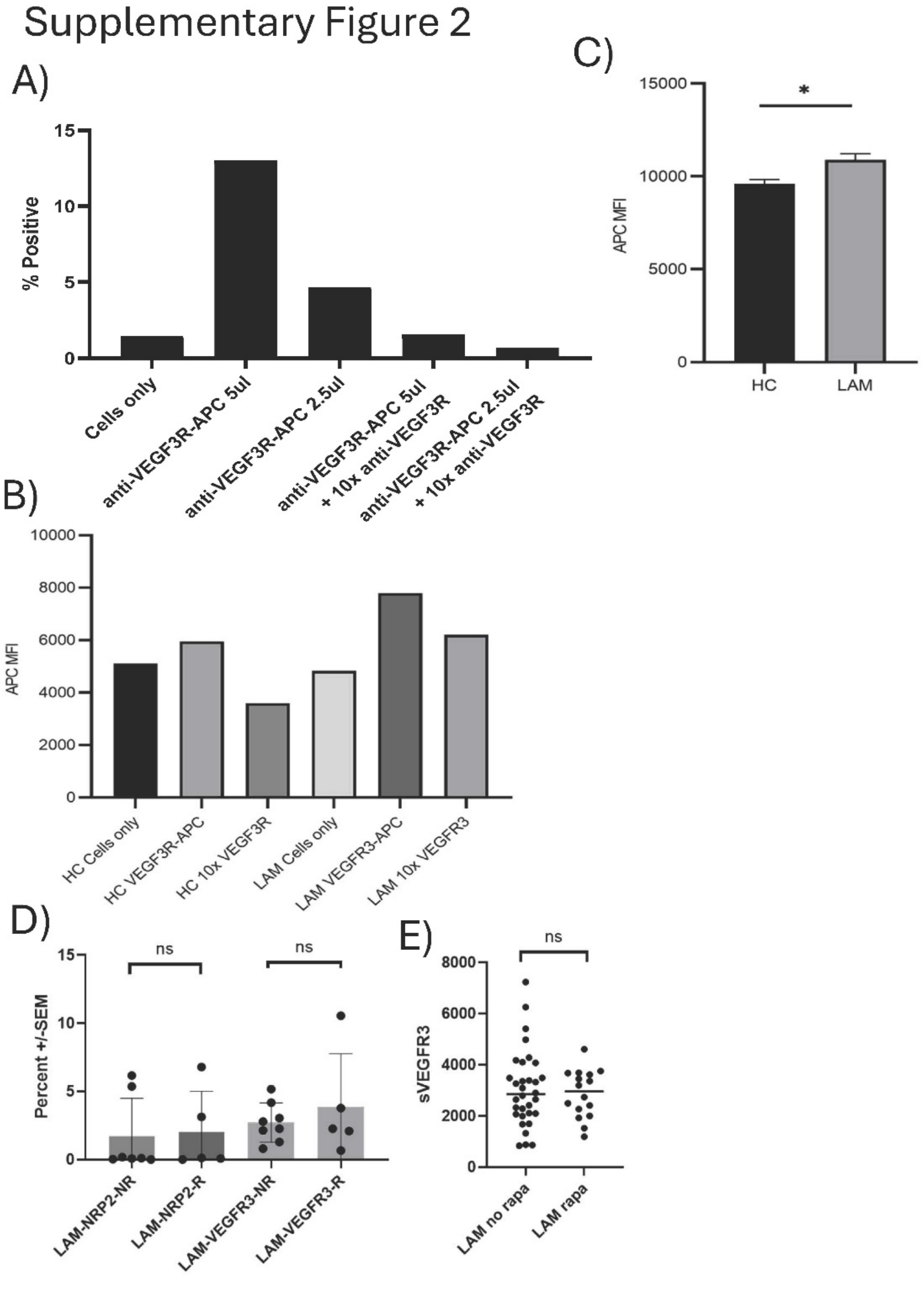

Supplemental Figure 2. Specificity of mouse anti-VEGFR3 antibody. A) NK cells were isolated by magnetic bead positive selection and stained for viability and then blocked with human Fcblock. Cells were either stained or not with a 10X excess of unlabeled with anti-VEGFR3 antibody for 25 minutes on ice in the dark. Cells were then stained with anti-VEGFR3-APC antibody for a further 25 minutes before washing and fixation. **A)** The percentage positive for each staining condition is shown. **B)** The median fluorescent intensity of the anti-VEGFR3-APC antibody is shown. **C)** HC and LAM cells were stained with anti-VEGFR3-APC antibody and MFI plotted, *P<0.05. **D)** plot of percent positive for NRP2 and VEGFR3 based on no rapamycin (NR) and rapamycin treatment (R). **E)** plot of effect of rapamycin on sVEGFR3 in serum on LAM patients. Bars represent mean ± SEM. **A–B**) representative plots, **C)** n= 3/group, **D**) n= 5–8/group, **E**) n=16–32.
